# Supplementary material for: Anterior-posterior gradient of plasticity in primate prefrontal cortex
Source: Nat Commun. 2018 Sep 17;9:3790. doi: 10.1038/s41467-018-06226-w (PMC6141600; doi:10.1038/s41467-018-06226-w)
Supplement: Supplementary file 1 — Supplementary Information [file 41467_2018_6226_MOESM1_ESM.pdf]

Anterior-posterior gradient of plasticity in primate prefrontal cortex

Riley et al.

Supplementary Information

SUPPLEMENTARY METHODS

Database of recordings used in different analyses

We collected data using some variations of tasks and stimulus sets, including sessions that used different shaped stimuli; sessions using a variable duration of the delay period; and sessions in which the sample stimulus was always a match. The selection of task and stimulus set was done prior to the onset of recordings and the properties of neurons recorded were not known ahead of time. We therefore used the largest available set that was appropriate for each analysis in order to increase statistical power. Our conclusions would not differ had we limited all analyses to a common dataset (S_5) but insufficient power would be available for some comparisons, in some areas.

In total, we recorded from 3908 neurons (set S_1) before and after training, while the monkeys were either passively viewing stimuli or performing the active task, across all task and stimulus sets (Posterior-dorsal region, n=182 neurons pre-training/211 neurons post-training; Mid-dorsal, n=756/690; Anterior-dorsal, n=359/222; Posterior-ventral, n=633/612; Anterior-ventral, n=122/121; see also Supplemental Table 1). We used this dataset in analyses testing responsiveness to the first stimulus in the trial, in Fig. 3A and S1.

A subset, S_2 , included 3704 neurons (95% of the total) that were tested with white squares, appearing at the nine stimulus locations of Fig. 1 and was used in the analyses comparing maximum firing rate, and spatial selectivity of neuronal responses to the first stimulus in the trial, in Fig. 3B and Fig. 3D-3G (Posterior-dorsal: Pre-training/post-training n=178/215 neurons; Mid-dorsal n=719/662; Anterior-dorsal n=313/212; Posterior-ventral n=610/586; Anterior-ventral n=92/117).

A subset of the preceding set, S_3, using delay periods of a fixed duration equal to 1.5 s, included 3508 neurons (90% of the total). These neurons were used in the analyses plotting PSTH across the trial in Fig. 2 and Mutual Information in Fig. 6 (Posterior-dorsal: Pre-training/post-training n=175/204 neurons; Mid-dorsal n=668/659; Anterior-dorsal n=298/212; Posterior-ventral n=502/581; Anterior-ventral n=92/117).

A subset of the S_2 set, S_4, included neurons tested with both match and nonmatch stimuli displayed during the sample period. We had 3132 neurons (80% of total) thus available, and these were used for the Match-Nonmatch difference analysis in Fig. 7 (Posterior-dorsal: Pre-training/post-training n=175/204 neurons; Mid-dorsal n=704/434; Anterior-dorsal n=305/122; Posterior-ventral n=551/441; Anterior-ventral n=92/104).

Finally a common subset of all preceding sets, S_5, included neurons tested with the stimuli at all spatial locations, with a fixed delay period duration equal to 1.5 s, and included both match and nonmatch stimuli. This set included 3040 neurons (78% of the total). This was used for the ROC analysis of Fig. 4 (Posterior-dorsal: Pre-training/post-training n=175/204 neurons; Mid-dorsal n=668/434; Anterior-dorsal n=298/122; Posterior-ventral n=502/441; Anterior-ventral n=92/104).

Additionally, when testing the spatial selectivity of delay period activity for figures 3C and 3H-3K, we only used neurons that exhibited significantly elevated activity during that period of the task (Posterior-dorsal: Pre-training/post-training n=34/77 neurons; Mid-dorsal n=174/253; Anterior-dorsal n=33/52; Posterior-ventral n=84/173; Anterior-ventral n=16/11). Similarly, for the analysis for the cue-period SI used in Supplementary Figure 4, we only used neurons that responded significantly during the cue period (Posterior-dorsal: n=65 neurons Pre-training/ 80 Post-training; Mid-dorsal: n=253/259; Anterior-dorsal: n=104/59; Posterior-ventral: n=66/123; Anterior-ventral: n=33/35). For the analysis examining correct and error trials in Supplementary

Figure 9, we used neurons during the post-training recordings with at least two error trials in the condition that elicited the best delay period response (Posterior-dorsal: n=134 neurons; Mid-dorsal n=127; Anterior-dorsal n=75; Posterior-ventral n=148; Anterior-ventral n=71). For measuring choice probability (Supplementary Figure 8), we distinguished between match/non-match preferring neurons, and we used neurons that had at least two error trials for the stimulus that evoked the best sample response for that condition (Non-match preferring neurons: Posterior-dorsal: n=54 neurons; Mid-dorsal n=32; Anterior-dorsal n=22; Posterior-ventral n=50; Anterior-ventral n=31; Match preferring neurons: Posterior-dorsal: n=49 neurons; Mid-dorsal n=51; Anterior-dorsal n=20; Posterior-ventral n=58; Anterior-ventral n=16).

Analysis based on same 4 monkeys tested before and after training

The results presented in the main text relied on all available data from 6 monkeys prior to training and 4 of these monkeys after they were trained in working memory tasks. To ensure that any changes between stages were not due to individual differences driven by the monkeys not tested after training, we repeated our analysis, restricting our sample to the same animals before and after training (Supplementary Figure 11). A total of 3482 neurons (89% of total) were available for this analysis. (Posterior-dorsal: Pre-training/post-training n=134/215 neurons; Mid-dorsal n=648/662; Anterior-dorsal n=237/212; Posterior-ventral n=597/586; Anterior-ventral n=74/117). The conclusions drawn from this dataset were essentially identical with what we report in the main text.

In terms of percentage of neurons activated during the delay period, a significant increase was present overall after training, which was greatest for the anterior-dorsal region. This region saw a significant increase in the percentage of neurons significantly responding during that epoch (pre-training 7%, post-training 24%; χ^2 test, $p=1.8\text{x}10^{-6}$).

In terms of firing rate (Supplementary Figure 11A), there were no differences between dorsal regions prior to training (1-way ANOVA, $F_{2,1016}=1.10$, $p=0.33$). In contrast, after training, the anterior-dorsal region had a higher firing rate than the other two dorsal regions (1-way ANOVA, $F_{2,1086}=5.40$, $p=0.005$ and Tukey post-hoc test, $p<0.05$). Higher firing rates were observed in the anterior ventral region both before (1-way ANOVA, $F_{(1,669)}=16.13$, $p=6.6\times10^{-5}$) and after training (1-way ANOVA, $F_{(1,701)}=10.6$, $p=0.001$).

The highest Selectivity Index values (Supplementary Figure 11B) were observed prior to training in the posterior-dorsal and mid-dorsal regions (1-way ANOVA, $F_{2,185}=15.14$, $p=8.07\times10^{-7}$). Following training, the selectivity in the anterior-dorsal region rose while the selectivity in the posterior-dorsal decreased. A 2-way ANOVA using training stage and dorsal regions as factors showed a significant interaction between the two ($F_{2,546}=11.08$, $p= 1.92\times10^{-5}$). There were no significant differences in the ventral regions prior to training (1-way ANOVA, $F_{1,93}=1.11$, $p=0.29$), but the posterior-ventral region had higher selectivity following training (1-way ANOVA, $F_{1,182}=4.12$, $p=0.04$).

Prior to training, no significant difference was present in terms of mutual information between dorsal areas (Supplementary Figure 11C) was observed (1-way ANOVA, $F_{2,967}=0.19$, $p=0.827$). Following training, mutual information increased significantly for all dorsal areas, however the increase was proportionally greater for the anterior dorsal areas (t-test, $p=0.018$, $p=9.7\times10^{-9}$, $p=5.0\times10^{-14}$, for the posterior, mid, and anterior areas, respectively). As a result, mean information values between dorsal areas was now significantly different (1-way ANOVA, $F_{2,1063}=16.4$, $p=9.8\times10^{-8}$). In the ventral regions, we saw a similar increase in Mutual Information after training, however the increase for the anterior-ventral region did not reach significance (t-test, $t_{183}=0.439$, $p=0.439$).

Finally, we examined the absolute difference between match and nonmatch responses between training stages (Supplementary Figure 11D). Prior to training, the posterior-dorsal region had a lower absolute difference than the mid-dorsal and anterior-dorsal (1-way ANOVA, $F_{2,991}$=3.82, p=0.02). This difference was not present when we included the data from all 6 monkeys. When we examined the effects of training using a 2-way ANOVA, we observed that there was a significant main effect of training stage ($F_{1,1751}$=40.23, p=2.9x10^{-10}) and interaction between training stage and dorsal region ($F_{2,1751}$=6.39, p=0.0017). There were no differences in match/nonmatch absolute difference response rates prior to training in the ventral region, but there were significant main effects of training stage ($F_{1,1153}$=19.92, p=8.9x10^{-6}) and interaction of training stage and ventral region ($F_{2,1153}$=13.03, p=3.2x10^{-4}).

In conclusion, analysis restricted to the same 4 subjects before and after training confirmed that the anterior areas exhibited the highest increase in the percentage of active neurons during the delay period, in firing rate during this period, mutual information, and in selectivity index. Restricting data to 4 monkeys also revealed a difference in |Match-Nonmatch| preference between dorsal areas prior to training, however this finding did not change that the highest increase was observed in the anterior dorsal area after training.

Analysis based of Neurons with Excellent Spike Isolation

In order to discount the possibility that changes were observed between training stages due to systematic differences in spike isolation, or levels of noise in recordings, we repeated our analysis based on a subset of neurons, for which excellent isolation was achieved, based on Signal to Noise Ratio of the spike waveform of greater than 5. A total of 2461 neurons were identified in this fashion (Posterior-dorsal: Pre-training/post-training n=141/146 neurons; Mid-dorsal n=543/373; Anterior-dorsal n=175/49; Posterior-ventral n=462/480; Anterior-ventral n=41/51). Firing rate changes before and after training in this set of neurons is shown in

Supplementary Figure 3. The results, including greater effects of training in the anterior and ventral areas, which extended in the baseline activity, mirror the changes documented in the entire sample of neurons (Fig. 2).

Recording Depth Analysis

Recording depths could be estimated approximately, based on the initial detection of neural activity as electrodes were advanced into the cortex and the relative distance traversed from that point to the recording site of each neuron. Our recordings targeted the supra-granular layers, based on anatomical expectations about neurons active during the delay period [1, 2]. Prior to training, mean recording depths in the dorsal areas were 0.45, 0.50, and 0.28 mm for the posterior, middle, and anterior region, respectively. These means included penetrations that descended into the principal sulcus, where greater depths do not necessarily imply deeper layers. After training, average depths also corresponded to the supra-granular layers: 0.38, 0.29, and 0.38 mm for the posterior, middle, and anterior region, respectively.

Depths of neurons in the ventral areas (which did not include sulci) were even more superficial. Prior to training, the mean depth of recordings was 0.34 and 0.25 mm for the posterior and anterior ventral PFC, respectively. After training the corresponding depths were 0.35 and 0.19 mm, for the two areas respectively.

Analysis of Individual-Monkey-Derived Means

We performed regression analyses on mean values obtained separately from each monkey, using the average of neuronal values from each monkey as a single observation, in order to ensure that results obtained by pooling all data were not skewed by data from 1-2 individuals. The dependence of neuronal measures on AP position determined in this fashion (Fig.

3H,I,J,K,P,Q,R,S) tended to agree with the results based from all data (Fig. 3D,E,F,G,L,M,N,O). No significant dependence of mean delay-period discharge rate on AP position was observed in dorsal areas prior to training (β_{pooled}= -0.2 spikes/percentiled-location), when all data were pooled together (Fig. 3D). A similar, negative slope ($\beta_{individual}$=-5.5) was also obtained from the individual-monkey-derived means (Fig. 3H). In contrast, a significant positive slope was observed after training from pooled data (β_{pooled}= 9.0, Fig. 3E) and a positive slope was obtained from individual-monkey data ($\beta_{individual}$=10.6, Fig. 3I). In the ventral prefrontal cortex, positive slopes were observed both before training (β_{pooled}= 5.8, $\beta_{individual}$=5.4, Fig. 3F/J) and after training (β_{pooled}= 8.0, $\beta_{individual}$=7.3, Fig. 3G/K).

Selectivity Index values also exhibited similar dependence on AP position when determined by pooling all data, or based on individual monkey derived means. Prior to training, a significant negative slope was present in dorsal prefrontal cortex based on pool data (β_{pooled}= -0.571, Fig. 3L). A very similar slope was obtained based on individual-monkey data ($\beta_{individual}$=-0.530, Fig. 3I). The slope of the regression curve significantly increased (became less negative) after training (β_{pooled}= -0.023, Fig. 3M), an increase that was mirrored in the individual monkey data ($\beta_{individual}$=-0.293, Fig. 3Q). In the ventral prefrontal cortex, a slightly positive dependence of Selectivity Index was present prior to training for both pooled and individual data (β_{pooled}= 0.103, $\beta_{individual}$=0.003, Fig. 3N/R). This changed to a negative slope after training (β_{pooled}= -0.210, $\beta_{individual}$=-0.361, Fig. 3F/J).

Similarly, differences between match and non-match responses showed the same dependence on AP position when obtained from pooled data, or from individual-monkey-derived means. Regressing the absolute difference between match and nonmatch responses |M-NM| on AP position produced slopes that were not significantly different than zero in the dorsal prefrontal cortex prior to training (β_{pooled}= 0.04, $\beta_{individual}$= -1.6). These were positive after training (β_{pooled}= 5.1, $\beta_{individual}$= 4.9). The same pattern of changes was observed in the ventral prefrontal

cortex, and with similar values between pooled and individual-monkey-derived data before training (β_{pooled}= 0.05, $\beta_{individual}$= -1.6) and after training (β_{pooled}= 8.0, $\beta_{individual}$= 11.2).

SUPPLEMENTARY DISCUSSION

Although our analysis relied on thousands of neurons sampled from six monkeys, it was not practical to sample all areas, in all animals. Data from the most anterior regions in particular were only drawn from a few animals. It is important to emphasize however, that the effects of training we reported in the anterior regions were consistent with previous reports in the literature that have described robust modulation of prefrontal neurons by task variables[3-7], which presumably come about after training. It was the relative lack of changes in the posterior areas after training (which were sampled densely, in multiple monkeys) that were responsible for the gradient of plasticity that we observed across the anterior-posterior axis of the prefrontal cortex (Fig. 3-7).

Why is the anterior prefrontal cortex more plastic after training in a spatial working memory task? Our neurophysiological experiments could not address this question, however, anatomical evidence points to an organization of inputs that funnels increasingly more complex information into anterior areas. The most posterior aspect of PFC, (posterior-dorsal in our study), receives sensory afferents [8-10], which allow a faithful representation of stimuli even in naïve animals. More anterior, hierarchically superior, auditory areas of the temporal lobe project to more anterior prefrontal subdivisions[11-13]. Face and color information appears to be concentrated in cortical patches, also suggestive of an anterior progression of more complex information[14]. Successively more anterior areas integrate inputs from posterior ones, appearing to have little specialization for stimulus features or sensorimotor mappings but have the capacity to be activated by higher order cognitive operations, suggesting a “rostral-caudal axis of cognitive control”[15]. Our experiments provide direct evidence that robust representation of stimulus information may emerge in the anterior prefrontal cortex following training, if it is necessary for execution of a cognitive task. A greater capacity for plasticity after training in a

cognitive task may also point to specialization of underlying cellular and molecular mechanisms[16], which may vary between prefrontal subdivisions[17] [18, 19].

SUPPLEMENTARY REFERENCES

1. Kritzer, M.F. & Goldman-Rakic, P.S. Intrinsic circuit organization of the major layers and sublayers of the dorsolateral prefrontal cortex in the rhesus monkey. *J. Comp. Neurol.* **359**, 131-143 (1995).
2. Pucak, M.L., Levitt, J.B., Lund, J.S. & Lewis, D.A. Patterns of intrinsic and associational circuitry in monkey prefrontal cortex. *J. Comp. Neurol.* **376**, 614-630 (1996).
3. Rigotti, M.*, et al.* The importance of mixed selectivity in complex cognitive tasks. *Nature* **497**, 585-590 (2013).
4. Mante, V., Sussillo, D., Shenoy, K.V. & Newsome, W.T. Context-dependent computation by recurrent dynamics in prefrontal cortex. *Nature* **503**, 78-84 (2013).
5. Lennert, T. & Martinez-Trujillo, J. Strength of response suppression to distracter stimuli determines attentional-filtering performance in primate prefrontal neurons. *Neuron* **70**, 141-152 (2011).
6. Asaad, W.F., Rainer, G. & Miller, E.K. Neural activity in the primate prefrontal cortex during associative learning. *Neuron* **21**, 1399-1407 (1998).
7. Freedman, D.J., Riesenhuber, M., Poggio, T. & Miller, E.K. Categorical representation of visual stimuli in the primate prefrontal cortex. *Science* **291**, 312-316 (2001).
8. Cavada, C. & Goldman-Rakic, P.S. Posterior parietal cortex in rhesus monkey: II. Evidence for segregated corticocortical networks linking sensory and limbic areas with the frontal lobe. *J. Comp. Neurol.* **287**, 422-445 (1989).
9. Petrides, M. & Pandya, D.N. Projections to the frontal cortex from the posterior parietal region in the rhesus monkey. *J. Comp. Neurol.* **228**, 105-116 (1984).
10. Romanski, L.M., Bates, J.F. & Goldman-Rakic, P.S. Auditory belt and parabelt projections to the prefrontal cortex in the rhesus monkey. *J. Comp. Neurol.* **403**, 141-157 (1999).
11. Romanski, L.M. & Goldman-Rakic, P.S. An auditory domain in primate prefrontal cortex. *Nat Neurosci* **5**, 15-16. (2002).
12. Barbas, H. General cortical and special prefrontal connections: principles from structure to function. *Annu Rev Neurosci* **38**, 269-289 (2015).
13. Romanski, L.M.*, et al.* Dual streams of auditory afferents target multiple domains in the primate prefrontal cortex. *Nature Neurosci.* **2**, 1131-1136 (1999).
14. Lafer-Sousa, R. & Conway, B.R. Parallel, multi-stage processing of colors, faces and shapes in macaque inferior temporal cortex. *Nat Neurosci* **16**, 1870-1878 (2013).
15. Petrides, M. Lateral prefrontal cortex: architectonic and functional organization. *Philos Trans R Soc Lond B Biol Sci* **360**, 781-795 (2005).
16. Kuboshima-Amemori, S. & Sawaguchi, T. Plasticity of the primate prefrontal cortex. *Neuroscientist* **13**, 229-240 (2007).
17. Garcia-Cabezas, M.A., Joyce, M.K.P., John, Y.J., Zikopoulos, B. & Barbas, H. Mirror trends of plasticity and stability indicators in primate prefrontal cortex. *Eur J Neurosci* **46**, 2392-2405 (2017).
18. McEwen, B.S. & Morrison, J.H. The brain on stress: vulnerability and plasticity of the prefrontal cortex over the life course. *Neuron* **79**, 16-29 (2013).
19. Laroche, S., Davis, S. & Jay, T.M. Plasticity at hippocampal to prefrontal cortex synapses: dual roles in working memory and consolidation. *Hippocampus* **10**, 438-446 (2000).

SUPPLEMENTARY FIGURES

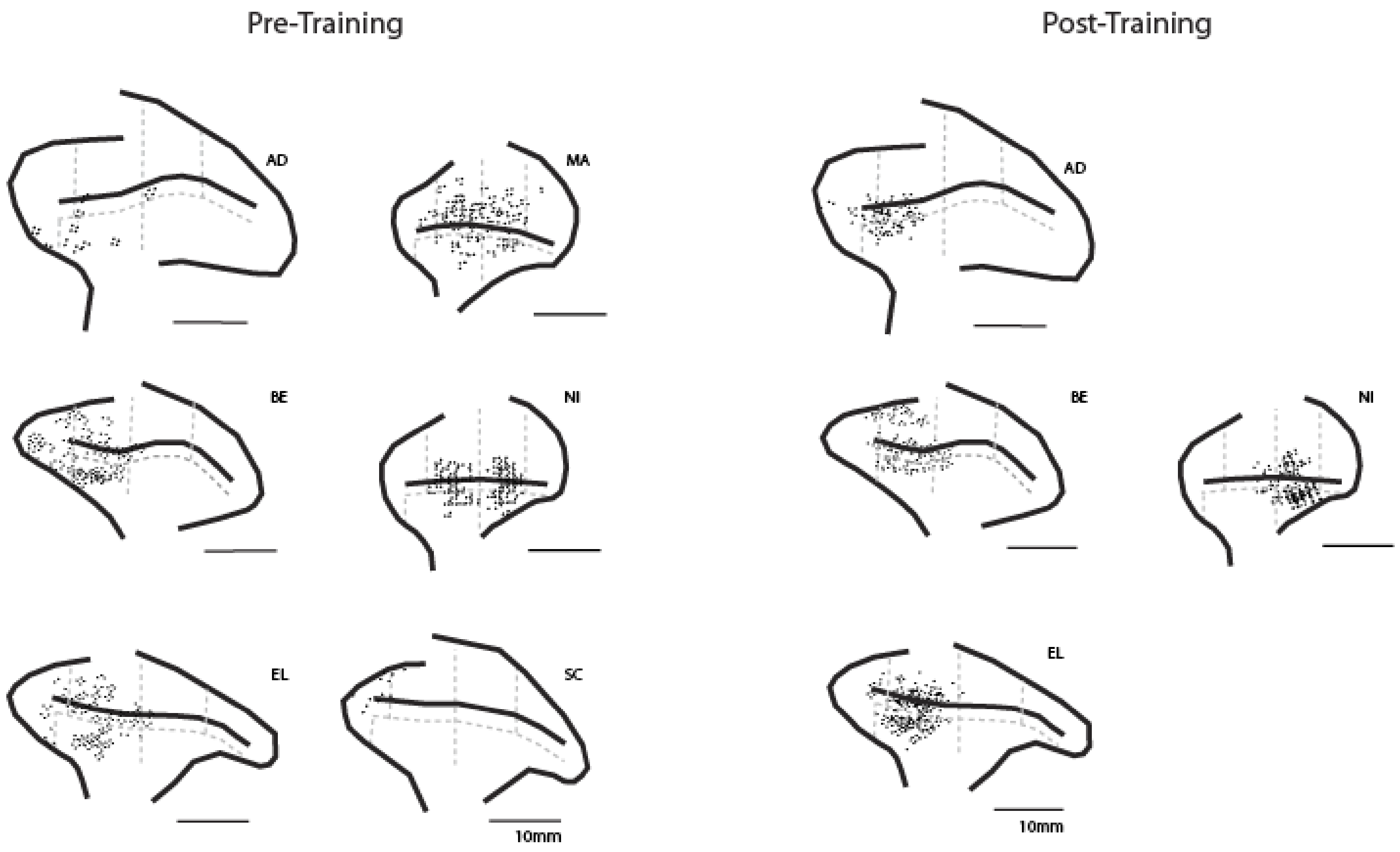

Supplementary Figure 1. Anatomical Localization. Electrode penetration maps for each individual monkey, before and after training. Black lines represent the arcuate and principal sulcus. Points represent electrode tracks, from which more than one neuron may have been recorded. Dashed gray lines represent the boundaries for each region.

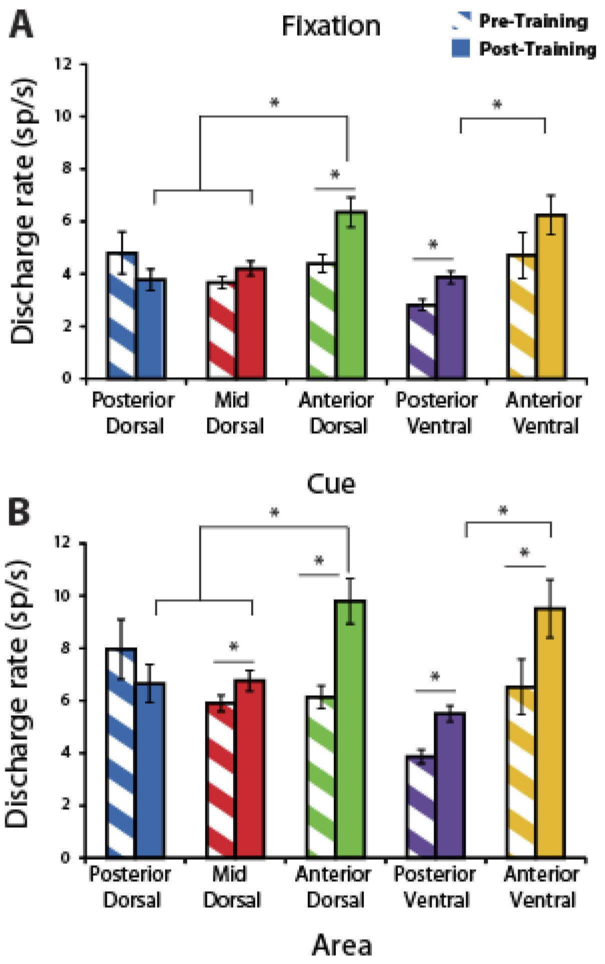

Supplementary Figure 2. Firing rate in fixation and cue periods. A. Bar graphs represent the mean firing rate during the fixation period of the task, for all neurons recorded in each area. Error bars represent the standard error of the mean calculated across all neurons. B. Mean firing rates during the cue period of the task. The best cue location was used for each neuron. Neurons are the same as Figure 3B (n=3908 total neurons).

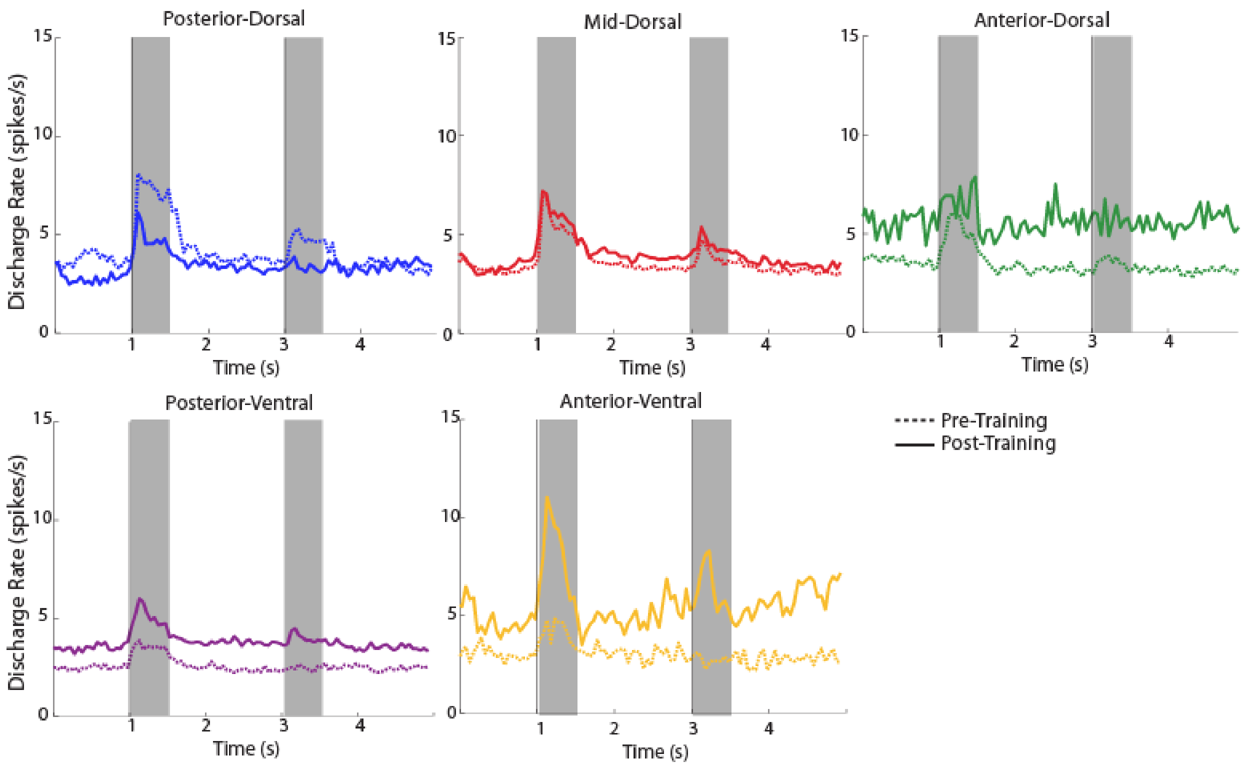

Supplementary Figure 3. Firing rate changes after training in neurons with SNR>5. Peri-stimulus Time Histogram depicting mean firing rate during the trial, based on each neuron's best cue location, before and after training. Conventions are the same in Fig. 2. Data are shown separately for each prefrontal region (Posterior-dorsal: Pre-training/post-training n=141/146 neurons; Mid-dorsal n=543/373; Anterior-dorsal n=175/49; Posterior-ventral n=462/480; Anterior-ventral n=41/51).

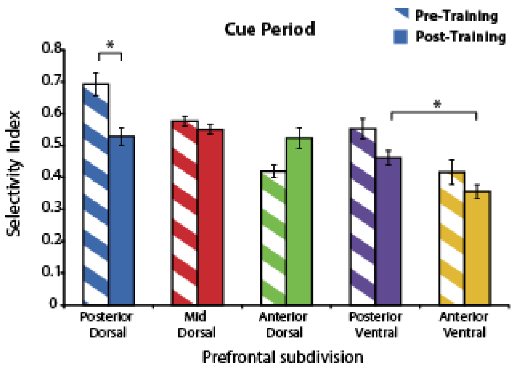

Supplementary Figure 4. Selectivity index during the cue period. Mean selectivity index values during the cue period before and following training for neurons responsive during the cue. Only neurons that responded significantly during the cue period were analyzed (Posterior-dorsal: n=65 neurons pre-training/ 80 post-training; Mid-dorsal: n=253/259; Anterior-dorsal: n=104/59; Posterior-ventral: n=66/123; Anterior-ventral: n=33/35). Error bars represent the standard error of the mean calculated across all neurons. Line diagrams represent significant differences between bars, calculated using a 1-way ANOVA (p<0.05).

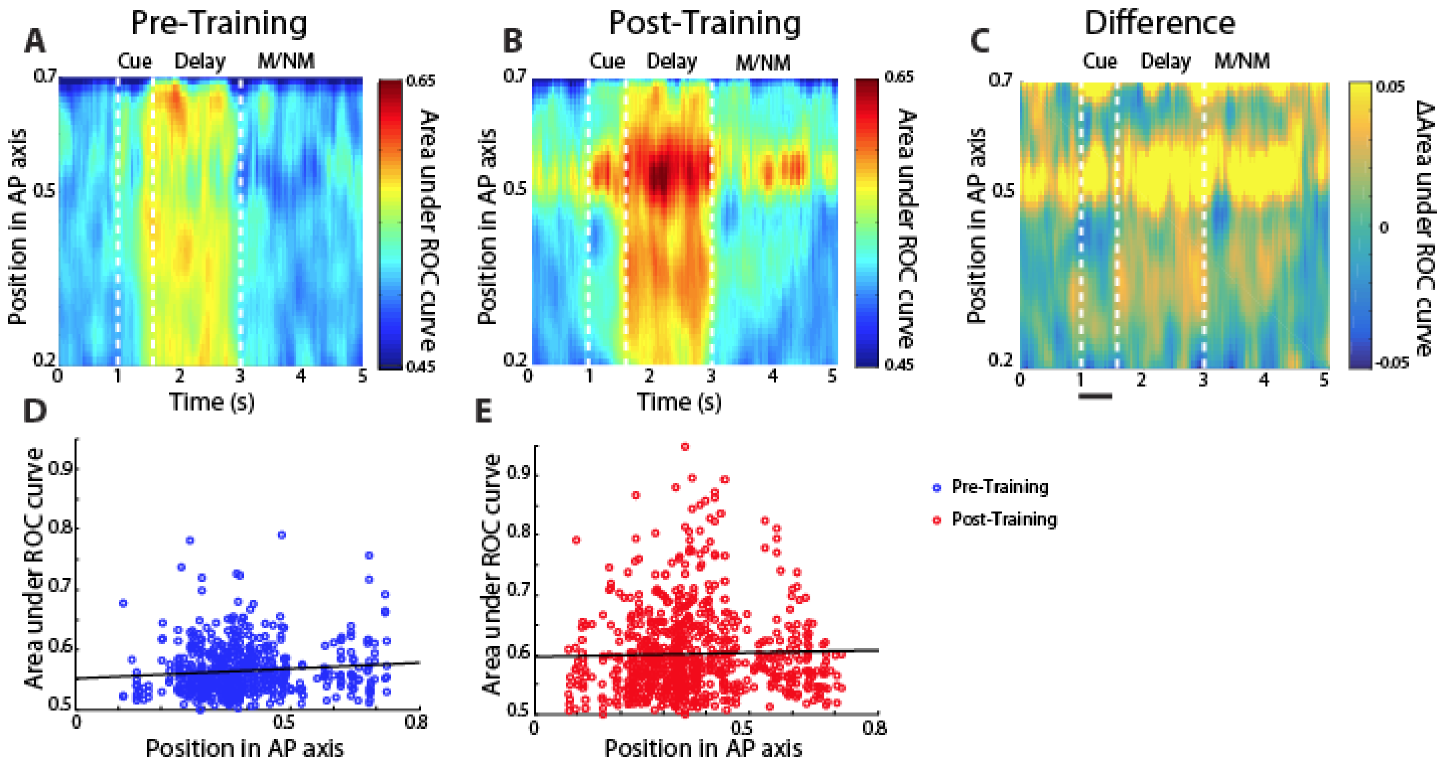

Supplementary Figure 5. Area under the ROC in the ventral PFC. A-B: ROC values are plotted along the Anterior-Posterior axis of the ventral prefrontal cortex, during the course of the trial, before (n=594) and after training (n=545). Conventions and analyses are the same as in Figure 4.

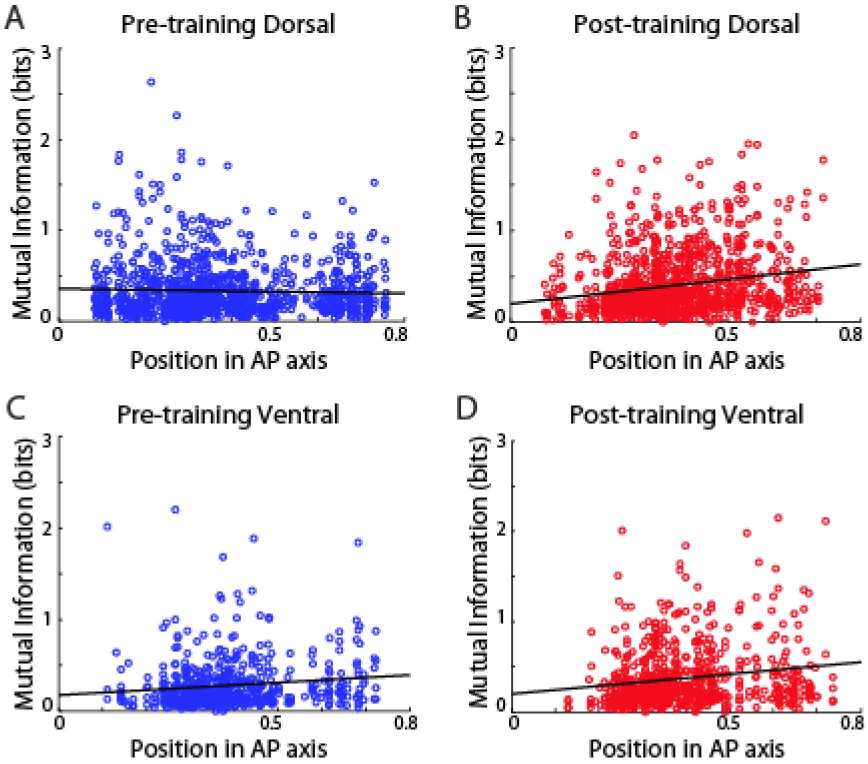

Supplementary Figure 6. Mutual Information Analysis in Cue Period. A. Mutual Information values based on spike counts in the cue presentation period are plotted along the Anterior-Posterior axis of the dorsal prefrontal cortex before and after training. Each point represents the Mutual Information value of one neuron, prior to training. Line represents linear regression. B. Mutual Information values in the ventral prefrontal cortex. C-D. Mutual information values after training. Sample of neurons is the same as in Figure 5 (n=3511 neurons).

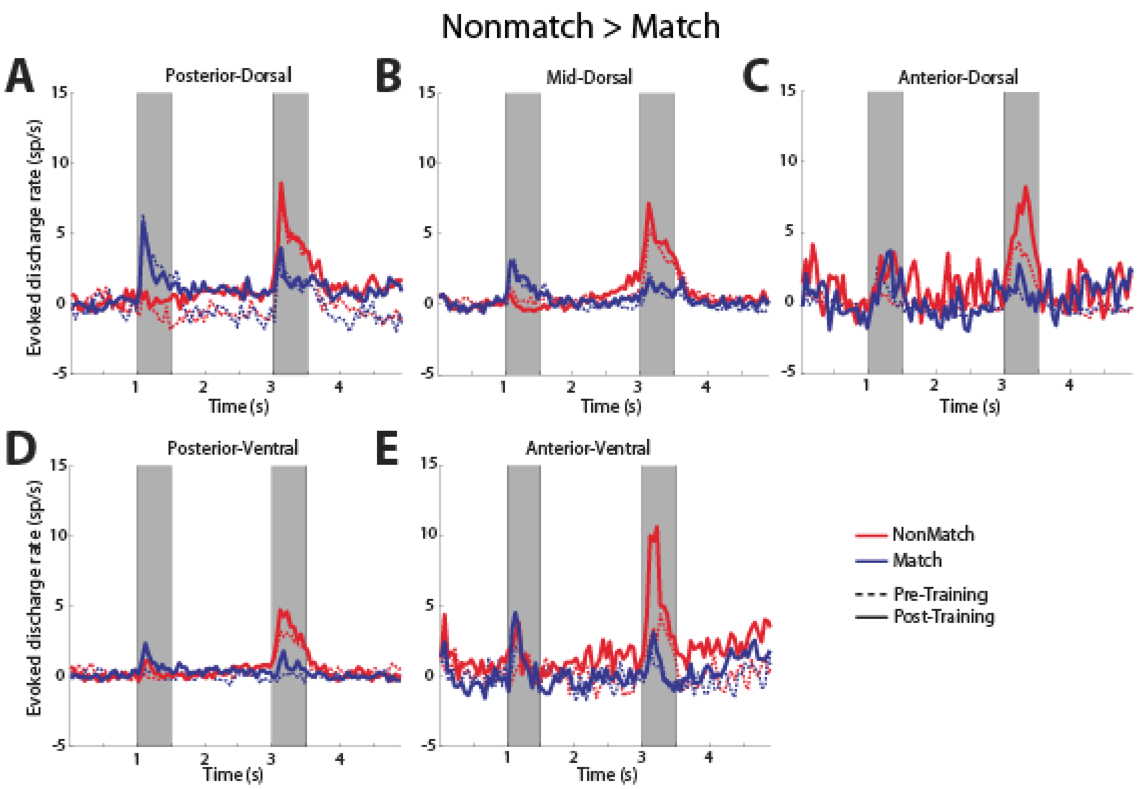
Nonmatch > Match
A
Posterior-Dorsal
B
Mid-Dorsal
C
Anterior-Dorsal
D
Posterior-Ventral
E
Anterior-Ventral
Evoked discharge rate (sp/s)
Time (s)
NonMatch
Match
Pre-Training
Post-Training

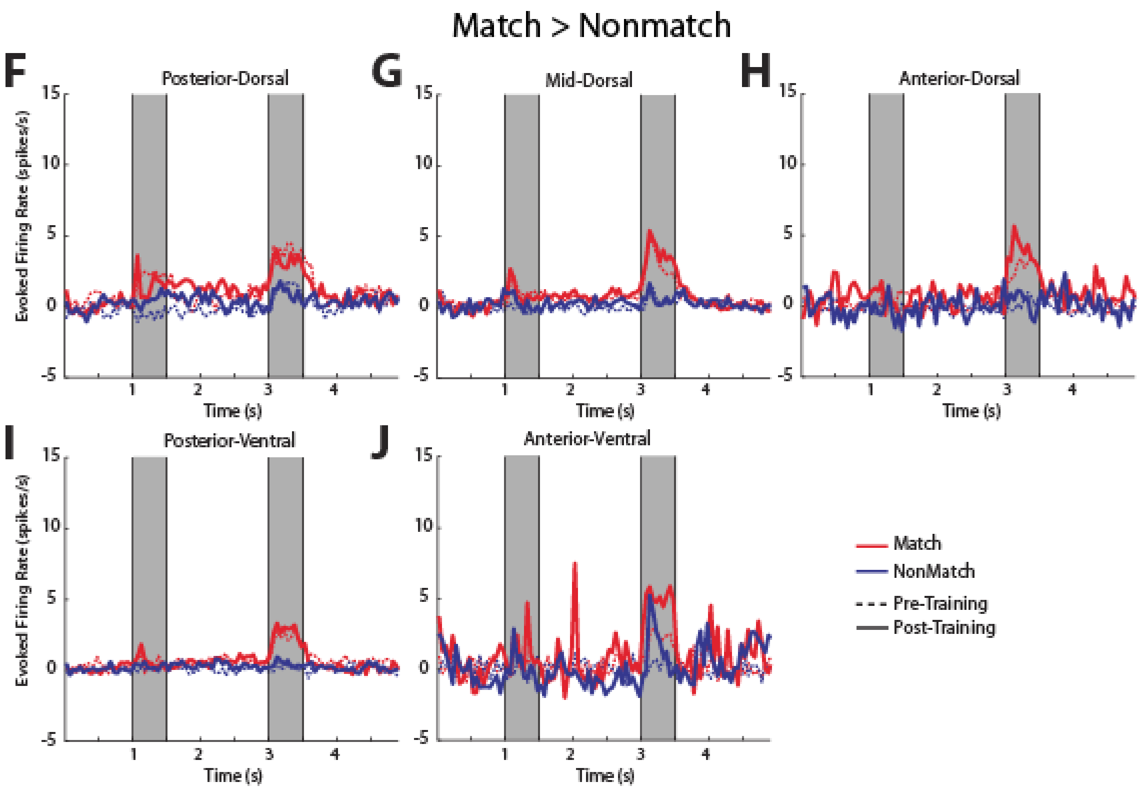
Match > Nonmatch
F
Posterior-Dorsal
G
Mid-Dorsal
H
Anterior-Dorsal
I
Posterior-Ventral
J
Anterior-Ventral
Evoked Firing Rate (spikes/s)
Time (s)
Match
NonMatch
Pre-Training
Post-Training

Supplementary Figure 7. Match-Nonmatch responses. A-E. Average firing rate difference between the responses of a stimulus presented as Nonmatch and as Match for neurons with overall greater responses to the Nonmatch. Nonmatch-match difference is depicted before training (dashed lines) and following training (solid lines). The shaded bars in each panel represents the cue and sample presentation periods. Posterior-dorsal: Pre-training/post-training n=175/208 neurons; Mid-dorsal n=704/434; Anterior-dorsal n=305/122; Posterior-ventral n=551/441; Anterior-ventral n=92/104. F-J. Average firing rate difference between the responses of a stimulus presented as a Match and as a Nonmatch for neurons with overall greater responses to the match. Pre-training/post-training n=175/208 neurons; Mid-dorsal n=704/434; Anterior-dorsal n=305/122; Posterior-ventral n=551/441; Anterior-ventral n=92/104.

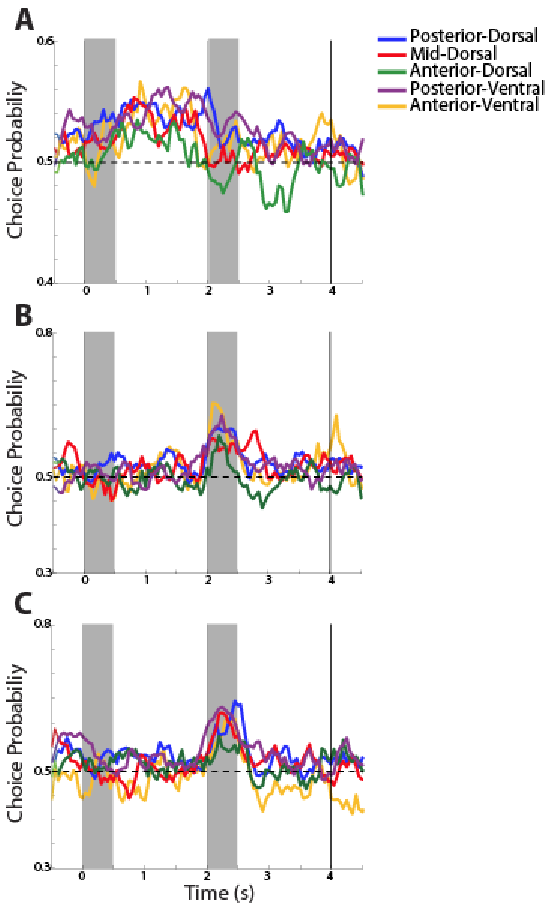

Supplementary Figure 8. Choice Probability. A. The mean area under the ROC curve is plotted for each prefrontal region, comparing the distribution of firing rates in correct and error trials (Choice Probability), after training. Location that elicited the best delay period of each neuron is used. Choice probability values from neurons with at least 2 error trials committed in this stimulus location are averaged (Posterior-dorsal: n=134 neurons; Mid-dorsal n=127; Anterior-dorsal n=75; Posterior-ventral n=148; Anterior-ventral n=71). B. Choice probability based on the best sample period, for neurons preferring nonmatch over match stimuli (Posterior-dorsal: n=54 neurons; Mid-dorsal n=32; Anterior-dorsal n=22; Posterior-ventral n=50; Anterior-ventral n=31). C. Choice probability based on the best sample period, for neurons preferring match over nonmatch stimuli (Posterior-dorsal: n=49 neurons; Mid-dorsal n=51; Anterior-dorsal n=20; Posterior-ventral n=58; Anterior-ventral n=16).

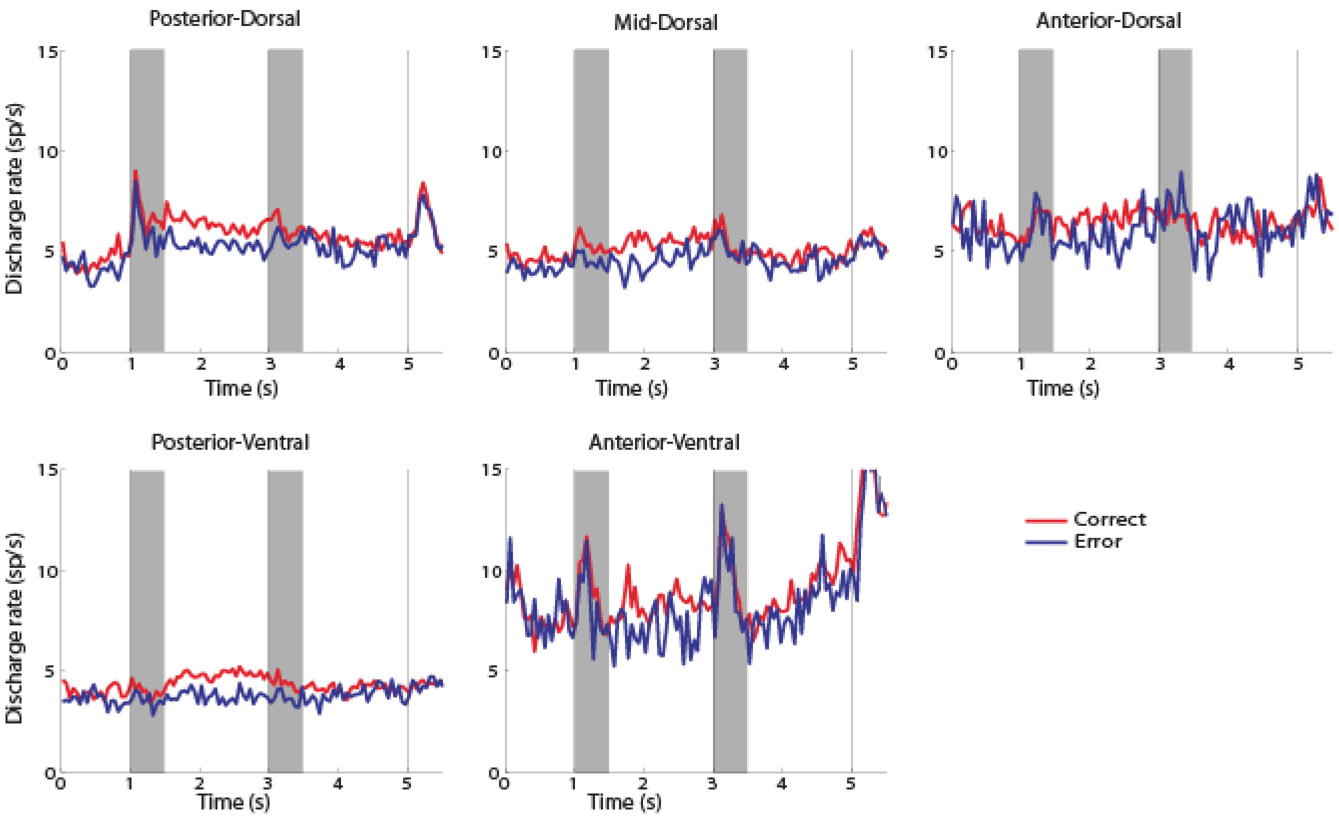

Supplementary Figure 9. Correct and Error Trials. Mean firing rate in correct and error trials are shown in each prefrontal subdivision. Location that elicited the best delay period of each neuron is used. Choice probability values from neurons with at least 2 error trials committed in this stimulus location are averaged (Posterior-dorsal: n=134 neurons; Mid-dorsal n=127; Anterior-dorsal n=75; Posterior-ventral n=148; Anterior-ventral n=71).

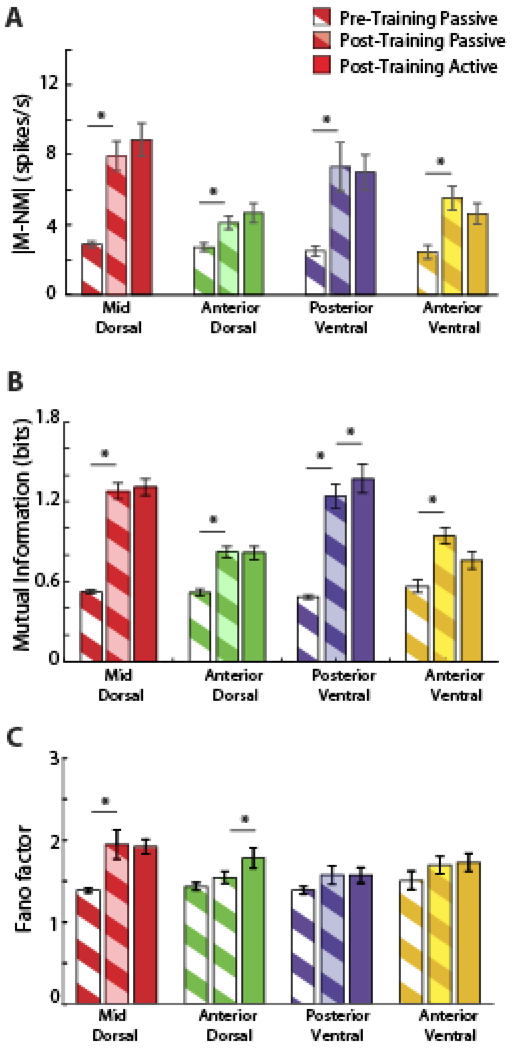

Supplementary Figure 10. Task Effects for all Neurons. A. Comparison of match and nonmatch responses in all neurons obtained prior to training, when all stimuli were presented passively, and after training, for all neurons tested both with the passive fixation and with the active working memory task (mid-dorsal: n=704 pre-training, n=129 post-training; anterior-dorsal: n=305 pre training, n=102 post-training; posterior-ventral, n=551 pre-training, n=42 post-training; anterior-ventral: n=92 pre-training, n=85 post-training). Conventions are the same as in Figure 8. Stars indicate significant differences in 1-tailed, t-test between pre-training passive and post-training passive measures, or in 1-tailed, paired t-test in post-training passive vs.

active comparisons. Pre-training data are the same as in Figure 7A. B. Mean Mutual Information values in each prefrontal region. C. Mean Fano factor values in each prefrontal region.

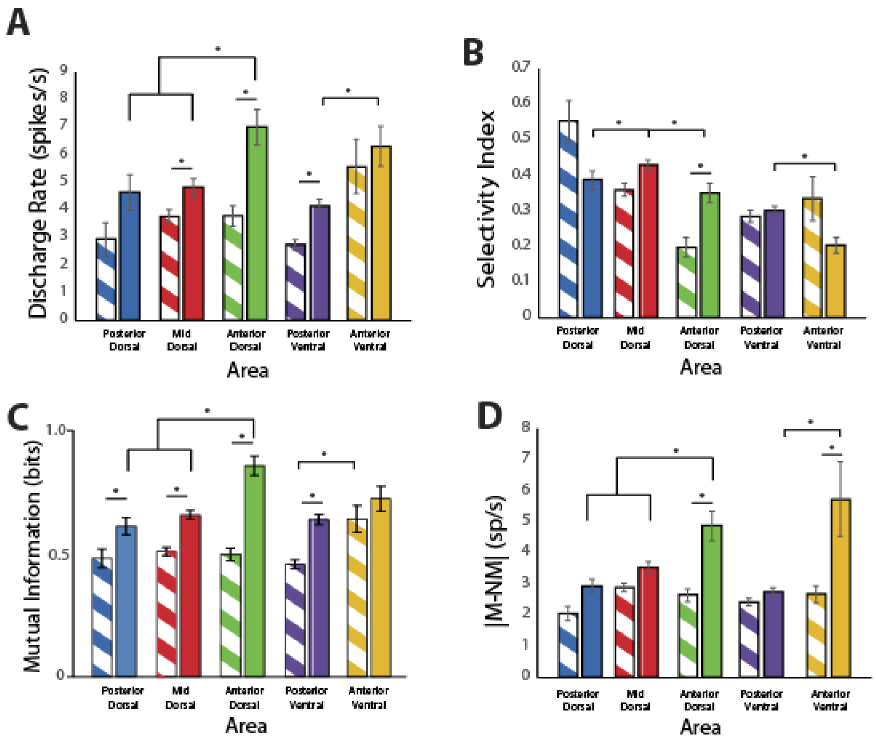

Supplementary Figure 11. Analysis Based on Same Monkeys. Comparison of pre-training and post-training data using only the same four animals that were trained in the working memory tasks. (A) Mean (and SEM) firing rate during the delay period (n=3482 neurons Posterior-dorsal: Pre-training/post-training n=134/215 neurons; Mid-dorsal n=648/662; Anterior-dorsal n=237/212; Posterior-ventral n=597/586; Anterior-ventral n=74/117.). (B) Mean value of selectivity index, among neurons responsive during the delay period. (C) Mean value of Mutual information. (D) Difference between firing rate of a stimulus appearing as a match and as a nonmatch.

**Pre-Training**																														
**Monkey**	ADR					BEN					ELV					SCR					MAN					NIN				
**Region**	PD	MD	AD	PV	AV	PD	MD	AD	PV	AV	PD	MD	AD	PV	AV	PD	MD	AD	PV	AV	PD	MD	AD	PV	AV	PD	MD	AD	PV	AV
**Neurons**	36	24	5	48	0	42	156	12	222	0	59	346	55	315	4	26	15	0	0	0	19	76	116	13	43	0	139	171	35	75
**Significant**	20	5	2	16	0	12	60	4	42	0	24	187	14	49	1	11	3	0	0	0	13	33	46	5	21	0	33	66	14	36
**Significant (Cue)**	16	2	0	12	0	7	44	4	19	0	23	166	14	23	1	10	1	0	0	0	11	28	41	4	20	0	23	64	12	31
**Significant (Delay)**	11	3	2	7	0	9	31	1	34	0	5	102	0	39	0	3	3	0	0	0	7	24	20	4	5	0	22	14	2	19
**Significant (Both)**	7	0	0	3	0	4	15	1	11	0	4	81	4	13	0	2	1	0	0	0	5	19	15	3	4	0	12	12	0	14
**Cue Period**																														
**Spatially Selective**	16	2	0	12	0	7	44	4	19	0	22	156	11	19	1	10	1	0	0	0	10	27	25	4	5	0	23	64	12	27
**Delay Period**																														
**Spatially Selective**	11	3	2	7	0	9	31	1	33	0	4	93	4	34	0	3	3	0	0	0	7	22	12	4	1	0	22	14	2	15
**Post-Training**																														
**Monkey**	ADR					BEN					ELV															NIN				
**Region**	PD	MD	AD	PV	AV	PD	MD	AD	PV	AV	PD	MD	AD	PV	AV											PD	MD	AD	PV	AV
**Neurons**	129	47	0	99	0	0	233	92	148	14	82	375	5	344	0											0	35	125	21	107
**Significant**	72	14	0	47	0	0	109	55	44	4	40	233	5	138	0											0	5	17	0	40
**Significant (Cue)**	58	9	0	39	0	0	79	45	15	2	23	183	4	74	0											0	4	11	0	34
**Significant (Delay)**	51	9	0	28	0	0	71	42	39	3	28	164	4	112	0											0	3	8	0	9
**Significant (Both)**	37	4	0	20	0	0	41	32	10	1	11	114	4	48	0											0	2	2	0	3
**Cue Period**																														
**Spatially Selective**	59	8	0	38	0	0	78	44	14	1	21	169	4	71	0											0	4	11	0	34
**Delay Period**																														
**Spatially Selective**	50	9	0	27	0	0	69	41	37	2	7	154	3	109	0											0	3	8	0	9

SUPPLEMENTARY TABLE 1

Supplementary Table 1: Sample Size. Prior to training, responses were recorded from 2052 prefrontal neurons in six monkeys during a passive fixation task (monkey AD – 113 neurons, BE – 432, EL – 779, MA – 268, NI – 419, SCR – 41 neurons). These six monkeys had no prior history of training in working memory tasks and were only required to view stimuli passively. Following training in a spatial working memory task, responses were recorded from 1856 neurons from four of these monkeys (monkey AD – 275 neurons, BE – 487 neurons, EL – 806 neurons, NI – 288 neurons).

Mean Age Pre-Training (yrs)					
	**Region**				
**Monkey**	PD	MD	AD	PV	AV
ADR	7.5	7.5	7.5	7.5	
BEN	7.4	7.4	7.5	7.5	
ELV	6.1	6.1	6.1	6.1	6.1
SCR	7.2	7.1			
MAN	5.5	6.7	6.7	6.0	7.2
NIN		6.2	6.1	6.2	6.0
**Average**	**6.7**	**6.8**	**6.8**	**6.7**	**6.5**

Mean Age Post-Training (yrs)					
	**Region**				
**Monkey**	PD	MD	AD	PV	AV
ADR	9.0	8.6		9.0	
BEN		9.5	9.7	9.6	9.6
ELV	7.5	7.7	6.8	7.7	
SCR					
MAN					
NIN		8.0	8.0	8.1	8.2
**Average**	**8.3**	**8.5**	**8.2**	**8.6**	**8.9**

Difference (yrs)					
	**Region**				
**Monkey**	PD	MD	AD	PV	AV
ADR	1.5	1.2		1.5	
BEN		2.0	2.2	2.1	
ELV	1.4	1.6	0.7	1.5	
SCR					
MAN					
NIN		1.9	1.9	1.9	2.2
**Average**	**1.5**	**1.7**	**1.6**	**1.8**	**2.2**

Supplementary Table 2: Monkey ages. The age of the monkey during recording of each neuron was determined, and then values were averaged separately for each subject, and prefrontal region. Mean age values obtained in this manner are reported for recordings obtained in the pre-training stage (top), and post-training stage (middle panel). Bottom line of each panel reports grand average of these age values. Difference in ages between pre- and post-training stages were also calculated, for those cases that recordings were available both before and after training (bottom panel).
